# Lack of nosocomial transmission to exposed inpatients and coworkers in an investigation of five SARS-CoV-2–infected healthcare workers

**DOI:** 10.1017/ice.2020.392

**Published:** 2020-08-03

**Authors:** Kwadwo Mponponsuo, Geneviève Kerkerian, Ranjani Somayaji, Bayan Missaghi, Joseph V. Vayalumkal, Oscar E. Larios, Byron M. Berenger, Matt Lauzon, Nicole McDonnell, John Conly

**Affiliations:** 1 Department of Medicine, Cumming School of Medicine, University of Calgary and Alberta Health Services, Calgary, Alberta, Canada; 2 Department of Microbiology, Immunology and Infectious Diseases, University of Calgary, Calgary, Alberta, Canada; 3 Department of Community Health Sciences, University of Calgary, Calgary, AB, Canada; 4 O’Brien Institute for Public Health, University of Calgary and Alberta Health Services, Calgary, AB, Canada; 5 Snyder Institute for Chronic Diseases, University of Calgary and Alberta Health Services, Calgary, AB, Canada; 6 Department of Pediatrics, Cumming School of Medicine, University of Calgary and Alberta Health Services, Calgary, Alberta, Canada; 7 Infection Prevention and Control, Alberta Health Services, Calgary, Alberta, Canada; 8 Department of Pathology and Laboratory Medicine, Cumming School of Medicine, University of Calgary and Alberta Health Services, Calgary, Alberta, Canada; 9 Alberta Public Health Laboratory, Alberta Precision Laboratories, Calgary, Alberta, Canada; 10 Workplace Health and Safety, Alberta Health Services, Calgary, Alberta, Canada

*To the Editor—*Clinical equipoise remains regarding the optimal protective measures and equipment to prevent nosocomial transmission risk of severe acute respiratory coronavirus virus 2 (SARS-CoV-2), the causative agent of coronavirus disease 2019 (COVID-19).^1^ Underpinning this question is debate regarding airborne versus droplet transmission of the virus. We evaluated 5 consenting healthcare workers (HCWs) who were diagnosed with community-acquired COVID-19 and who had interacted with patients and other HCWs (n = 72) while symptomatic or presymptomatic in the Calgary Health Zone of Alberta Health Services between March 1 and April 15, 2020.

Approval from the University of Calgary Ethics Committee (no. REB20-0510) was obtained to conduct interviews following verbal consent using a standardized case report form and questionnaire. Index HCWs and their patient and coworker exposures (Supplementary Table S1 online) were identified through databases and tracing with the infection prevention and control and occupational health departments. We utilized a risk assessment adapted from previously published guidance for contact tracing. We deemed close contact an interaction of >15 minutes at a distance of <1 m.^2^ Those exposed to the index HCWs were followed for 30 days for compatible SARS-CoV-2 infection symptom development. SARS-CoV-2 test results were obtained on exposed individuals who developed symptoms. Testing for SARS-CoV-2 was performed using a multiplex reverse-transcriptase real-time polymerase chain reaction (RT-PCR) targeting the envelope and the RNA-dependent RNA polymerase encoding regions (E and RdRp genes).^3^

All 5 of the HCWs (ie, HCWs A–E) had tested positive for SARS-CoV-2 by RT-PCR with E gene cycle threshold (Ct) values between 10.9 and 30.2 via nasopharyngeal or deep nasal swab and had symptoms prior to or on the day they worked (Supplementary Table S2 online). HCWs B and E worked 2 days while symptomatic, and the remainder worked 1 day. HCW A developed symptoms of mild nasal and sinus congestion the day of her shift; HCW B developed a sore throat 5 days prior to the day he worked; HCW C had a fever and cough develop while at work; HCW D developed fever, chills, and rhinorrhea the evening following her shift; and HCW E had sneezing, headache, fatigue, and sore throat on the days she worked.

Between the index cases, a total of 39 HCWs (Supplementary Table S1 online) were exposed (range, 6–12 per HCW). All index cases interacted with at least 5 other HCWs at a distance of < 1 m for >15 minutes. Of the exposed HCWs who underwent testing (n = 16), none tested positive for SARS-CoV-2 in the follow-up period. Notably, HCW B was undergoing training and partnered with another HCW for 2 hours, providing direct patient care.

In total, 33 patients were exposed to the index cases (range, 2–24) (Supplementary Table S1 online). HCW E did not have any patient exposure. Of the patients exposed to HCW A, 20 of 24 (83%) were deemed close contacts. All of the patients of HCWs B, C, and D were exposed for >15 minutes at <1 m distance for the described interactions. Only HCW C and D wore a mask for all of their patient interactions (n = 6). Of 22 patients who underwent SARS-CoV-2 testing, none had a positive test result. Of the remaining 11 exposed patients, none developed symptoms or sought care in the follow-up period. No outbreaks were identified in the clinical areas in which the index cases worked.

Transmission risk is believed to be higher early in the disease, with lower Ct values on RT-PCR correlating to higher viral loads prior to the onset of symptoms.^4^ After the development of symptoms, transmission risk decreases corresponding to higher Ct detection values. In our study, low Ct values were seen in 4 of 5 index cases, suggesting high viable viral loads sufficient for transmission. However, transmission may have been mitigated by the consistent hand hygiene by all index HCWs. Consistent hand hygiene has previously been recognized as a protective factor against SARS and influenza.^5^ In addition, most respiratory pathogens are spread via direct contact, droplets, and fomites,^6^ and transmission to HCWs has been associated with lapses in hand hygiene as opposed to the lack of N95 mask use.^7^ Poor adherence to proper hand hygiene has been associated with the spread of SARS-CoV-2 to HCWs despite their use of other PPE.^8^

Our study has limitations, including a sample size of only 5 HCWs, and we did not directly observe PPE use by the HCWs. Nonetheless, there were a relatively large number of high-risk exposures (n = 63 of 72; 87.5% of total exposures). Secondly, the findings are subject to recall bias because we interviewed the index HCWs 2 weeks following their positive test results. Recall bias was minimized by examining multiple data sources for both index cases and exposed persons. Lastly, not all exposed individuals underwent testing following their exposures; however, none developed symptoms that would have warranted them to be tested at the time based on public health recommendations. Given the close follow-up and high testing rates, however, any missed symptomatic infections in this cohort were highly unlikely.

We did not identify any transmission events in multiple high-risk exposures from 5 COVID-19 HCWs to either patients or other providers. This finding is consistent with other studies where no transmission events occurred despite high-risk exposures in a hospital setting.^9,10^ It provides supporting data regarding the use of diligent hand hygiene and, in 2 cases, surgical mask use in routine patient management to mitigate SARS-CoV-2 transmission events. Our findings are inconsistent with airborne transmission with the lack of transmission to those exposed and are best explained by mitigations directed to a droplet or contact mode of transmission.

## Supplementary material

For supplementary material accompanying this paper visit http://dx.doi.org/10.1017/ice.2020.392. click here to view supplementary material

## References

[1] Eissenberg T , Kanj SS , Shihadeh AL. Treat COVID-19 as though it is airborne: it may be. AANA J 2020;88(3):29–30. 32347791

[2] Phin N. Expert interview: What is contact tracing? Public Health England website. https://publichealthmatters.blog.gov.uk/2020/02/13/expert-interview-what-is-contacttracing Published February 13, 2020. Accessed July 26, 2020.

[3] Berenger BM , Fonseca K , Schneider AR , et al. Sensitivity of nasopharyngeal, nasal, and throat swab for the detection of SARS-CoV-2. medRxiv 2020. doi. 10.1101/2020.05.05.20084889.

[4] Bullard J , Dust K , Funk D , et al. Predicting infectious SARS-CoV-2 from diagnostic samples. Clin Infect Dis 2020. doi: 10.1093/cid/ciaa638. PMC7314198 32442256

[5] Cowling BJ , Chan KH , Fang VJ , et al. Facemasks and hand hygiene to prevent influenza transmission in households: a cluster randomized trial. Ann Intern Med 2009;151:437–446. 19652172 10.7326/0003-4819-151-7-200910060-00142

[6] Kutter JS , Spronken MI , Fraaij PL , et al. Transmission routes of respiratory viruses among humans. Curr Opin Virol 2018;28:142–151. 29452994 10.1016/j.coviro.2018.01.001 PMC7102683

[7] Jin Y , Huang Q , Wang Y , et al. Perceived infection transmission routes, infection control practices, psychosocial changes, and management of COVID-19 infected healthcare workers in a tertiary acute care hospital in Wuhan: a cross-sectional survey. Mil Med Res 2020;7:24. 32393381 10.1186/s40779-020-00254-8 PMC7211983

[8] Ran L , Chen X , Wang Y , et al. Risk factors of healthcare workers with coronavirus disease 2019: a retrospective cohort study in a designated hospital of Wuhan in China. Clin Infect Dis 2020 Mar 17 [Epub ahead of print]. doi: 10.1093/cid/ciaa287. PMC7184482 32179890

[9] Wong SC , Kwong RT , Wu TC , et al. Risk of nosocomial transmission of coronavirus disease 2019: an experience in a general ward setting in Hong Kong. J Hosp Infect 2020;105:119–127. 32259546 10.1016/j.jhin.2020.03.036 PMC7128692

[10] Wendt R , Nagel S , Nickel O , et al. Comprehensive investigation of an in-hospital transmission cluster of a symptomatic SARS-CoV-2–positive physician among patients and healthcare workers in Germany. Infect Control Hosp Epidemiol 2020 Jun 3 [Epub ahead of print]. doi: 10.1017/ice.2020.268. PMC7298077 32489162

