# Supplementary material for: Lack of nosocomial transmission to exposed inpatients and coworkers in an investigation of five SARS-CoV-2–infected healthcare workers
Source: Infect Control Hosp Epidemiol. 2020 Aug 3:1–2. doi: 10.1017/ice.2020.392 (PMC7445459; doi:10.1017/ice.2020.392)
Supplement: Supplementary file 1 [file S0899823X2000392Xsup001.docx]

**Supplementary Table S1: Exposed Healthcare Workers/Patients and Exposure Details**

| **HCW** | **# of HCWs/**  **# of patients** | **Duties related to other HCWs/ patients** | **>15 minutes spent with HCWs and patients** | **Distance <1m with HCWs and**  **patients** | **Mask use around HCWs/ patients** | **Hand hygiene HCW/ patient interactions** | **COVID-19 Test (# tested) HCWs/patients** | **Number of Positive COVID-19 Tests HCW/patients** |
| --- | --- | --- | --- | --- | --- | --- | --- | --- |
| A | 12/24 | Conjoint patient care, handover, sitting side by side at a computer station, worked with security guard to help constrain a patient, eating lunch together/    vital signs assessment, insertion and removal of IVs, blood draws, medication administration, restraining  accompanying to imaging, mobility assistance | 10/12 and  20/24 | 10/12  and  20/24 | Not utilized for any interactions for either | Microsan™ for all interactions for both  Time spent on hand hygiene 10-20 seconds each time | 8/14 | 0/0 |
| B | 6/3 | Conjoint patient care, eating lunch together, handover, running list, charting, patient rounds/  admission to the ward, vital signs assessment, bedside work otherwise not specified, starting IVs, medication administration | 6/6  and  3/3 | 6/6  and  3/3 | Not utilized for any interactions  for either | Microsan™ for all interactions.  Time spent on hand hygiene about 10 – 20 seconds each time | 1/3 | 0/0 |
| C | 7/4 | Running patient list, patient handover, shared computer for charting, eating lunch together, assistance with dressing changes  vitals assessment, dressing changes, medication administration | 4/7  and  4/4 | 5/7  and  4/4 | Not utilized for any interactions with HCWs/  surgical mask for all patient interactions | Microsan™ for all interactions.  Time spent on hand hygiene 15 – 25 seconds each time | 4/4 | 0/0 |
| D | 6/2 | Conjoint patient care, handover, assisting with diagnostic imaging procedure, shared computer space, charting, eating lunch/  procedure explanation, medication administration | 6/6  and  2/2 | 6/6  and  2/2 | Surgical mask for one of the 6 HCW  interactions.  No mask used for the rest of the interactions/  surgical mask for all patient interactions | Soap and water used for all hand hygiene with surgical cleaning technique utilized (hands to elbows cleaned) ~ 20 – 30 seconds per interaction both HCW and patient interactions  Paper towel used to dry hands. | 0/1 | 0/0 |
| E | 8/0 | Patient chart reviews, discussing patient care with other HCWs, coffee/tea break, phone calls to patients with other HCWs nearby/  N/A | 8/8  and  N/A | 6/8  and  N/A | Not utilized for any interactions/  N/A | Microsan™ for all interactions.  Time spent on hand hygiene less than 20 seconds each time/  N/A | 3/  N/A | 0/  N/A |

Microsan^TM^ – 72% alcohol-based sanitizer

**Supplementary Table S2. Index HCW Characteristics**

| HCW | **Age** | **Sex** | **Role** | **Location** | **Suspected Cause of Initial SARS-CoV-2 Acquisition** | **Date(s) of Exposure Events** | **Fever (Tmax °C)** | **Symptoms** | **COVID-19 test date – result**  **E gene / RNAdPol**  **Cycle threshold** |
| --- | --- | --- | --- | --- | --- | --- | --- | --- | --- |
| A | 54 | F | RN | ER | Travel to Paris, France. Returned March 6, 2020 | March 10^th^, 2020 | N | Fatigue  Sore throat, cough, myalgias, sinus congestion, anosmia, dysgeusia | March 13^th^, 2020 - Positive  24.4/27.9 |
| B | 25 | M | RN | CCU | Partner with symptoms consistent with SARS-CoV-2 March 17, 2020 | March 21^st^ and 22^nd^, 2020 | Y (38.5) | Fatigue, headache, cough, nasal congestions, dysgeusia, anosmia, sneezing | March 24^th^, 2020 – Positive  13.7/17.9 |
| C | 35 | F | LPN | Hemodialysis Unit | Church service on March 8, 2020 with exposure to known case | March 15^th^, 2020 | Y (38.2) | Fatigue, myalgias, anosmia, dysgeusia | March 17^th^,2020 – Positive  16.4/19.7 |
| D | 36 | F | RN | Diagnostic Imaging Radiology | Partner with symptoms consistent with SARS-CoV-2 April 1^st^, 2020 | April 6^th^, 2020 | Y (N/A) | Fatigue, cough, nasal congestion, rhinorrhea, anosmia, dysgeusia, nausea | April 7^th^, 2020 – Positive  10.96/13.95 |
| E | 36 | F | Dietician | Outpatient clinic | Unclear. Grocery store visit March 26, 2020 suspected by Public Health | April 1^st^ and 2^nd^, 2020 | Y (38.0) | Fatigue, headache, sore throat, nasal congestion, sneezing, cervical lymphadenopathy | April 3, 2020 – Positive  30.62/33.88 |

RN = registered nurse

LPN = licensed practical nurse

ER = emergency room

CCU = coronary care unit

Tmax = maximum temperature
